# Supplementary material for: Using computed tomography to evaluate proper chest compression depth for cardiopulmonary resuscitation in Thai population: A retrospective cross-sectional study
Source: PLoS One. 2023 Feb 3;18(2):e0279056. doi: 10.1371/journal.pone.0279056 (PMC9897514; doi:10.1371/journal.pone.0279056)
Supplement: Attachment — Submitted filename: Response to reviewers.docx [file pone.0279056.s002.docx]

Dear Editor,

Thank you very much for giving us the opportunity to revise our manuscript. We have responded to all questions and comments as described below; if any further unclear, please do let us know.

Sincerely,

Phatthranit Phattharapornjaroen

Corresponding author

**Response to reviewers**

**#1. Is the manuscript technically sound, and do the data support the conclusions?**

**The manuscript must describe a technically sound piece of scientific research with data that supports the conclusions. Experiments must have been conducted rigorously, with appropriate controls, replication, and sample sizes. The conclusions must be drawn appropriately based on the data presented.**

**Reviewer #1: Partly**

**Reviewer #2: Yes**

***Answer:*** *To add more information to our study, we have included sample size estimation and further statistical analysis parts as described on pages 6,7 Line 117-130. In addition, we have also added more conclusions and suggestions which relevant to the objective and our result on pages 8, lines 156-157, and on pages 10, lines 199-201.*

**#2. Has the statistical analysis been performed appropriately and rigorously?**

**Reviewer #1: No**

**Reviewer #2: Yes**

***Answer:*** *For more rigorous statistical analysis, we performed further analysis by controlling for possible confounders (i.e., age and sex), which might affect the primary outcomes. Revised statistical part and results were on page 8, lines 156-157, and Table 4., subsequently.*

**#3. Have the authors made all data underlying the findings in their manuscript fully available?

The** [**PLOS Data policy**](http://www.plosone.org/static/policies.action#sharing) **requires authors to make all data underlying the findings described in their manuscript fully available without restriction, with rare exceptions (please refer to the Data Availability Statement in the manuscript PDF file). The data should be provided as part of the manuscript or its supporting information, or deposited to a public repository. For example, in addition to summary statistics, the data points behind means, medians and variance measures should be available. If there are restrictions on publicly sharing data—e.g. participant privacy or use of data from a third party—those must be specified.**

**Reviewer #1: No**

**Reviewer #2: Yes**

***Answer****: We have provided the raw data in the supplementary file attached to this revised submission.*

**4. Is the manuscript presented in an intelligible fashion and written in standard English?

PLOS ONE does not copyedit accepted manuscripts, so the language in submitted articles must be clear, correct, and unambiguous. Any typographical or grammatical errors should be corrected at revision, so please note any specific errors here.**

**Reviewer #1: Yes**

**Reviewer #2: Yes**

***Answer****: Thank you for the reviewers’ comments.*

**#5. Review Comments to the Author

Please use the space provided to explain your answers to the questions above. You may also include additional comments for the author, including concerns about dual publication, research ethics, or publication ethics. (Please upload your review as an attachment if it exceeds 20,000 characters)**

**Reviewer #1: The depth of chest compressions depends on the rescuer's BMI anywhere in the world. My brother-in-law (Thai nurse) weighs 78 kg and 174 centimeters tall, he certainly has no problem make chest compression 5 -6 centimeters deep a European patient. Much depends on the difference in weight between the rescuer and the victim also in the Caucasians and
Asians populations.**

***Answ****er: We agreed with your comments that the factor associated with chest compression depth was the rescuers’ BMI. And there were several studies mentioned on this topic [1]. However, our research question focused on whether the recommendation depth from the USA and Europe regions were appropriate for Asian patients, who may have different sizes of anatomical parameters. In addition, we examined whether too deep or too shallow compression affects the quality of compression or internal organ injury.*

*References*

Lin C-C, Kuo C-W, Ng C-J, Li W-C, Weng Y-M, Chen J-C. Rescuer factors predict high-quality CPR—a manikin-based study of health care providers. Am J Emerg Med. 2016;34: 20–24. doi:10.1016/j.ajem.2015.09.001

**#Reviewer #2: This is an interesting retrospective study regarding the distribution of heart compression fraction among different sex and BMI in Thai population. The conclusion is that the recommended depth of chest compression during cardiac arrest (5-6 cm) is validated (with the simulated chest ct scan model in object) also in Thai population.

The article is well written in sound English, easy to read, and the conclusions stem clearly from data and discussion. References are coherent with the text.**

***Answer****: Thank you for the reviewer’s comments and suggestions to improve the article*.

**#Some minor issues come to mind:
- "Currently, computed tomography (CT) scan with contrast of chest was used to simulate heart compression fraction (HCF), which is the proportion of the heart compressed by CC [15], and HCF was subsequently mentioned in several studies to demonstrate high-quality CC during CPR in various populations [16,17]" this section could be expanded a little to explain clearly the link between heart compression fraction and high quality CPR.**

***Answers*** *According to the reviewer’s suggestion, we have added more information on page 4, lines 64-69*

**#- However this has been correctly addressed by the authors, it is to be noted that the population enrolled in the study may not be representative of the general population nor of the population most affected by cardiac arrest in Thailand (most notably only 3.3% of patients were admitted for cardiac illness). If there were any patients affected by cardiac arrest in the population in study, it may be reasonable to underline this data (otherwiseif patients resuscitated form cardiac arrest were excluded, absent or very few, then it should be disclosed as it is a significant limitation).**

***Answers*** *We agree with the reviewer’s comment, and we have added this limitation on pages 9,10, lines 188-192.*

**#- The notion that "although lower HCF was found in the obese groups, sufficient HCF was generated at 60 mm CC depth" may be included in the conclusions, since it may yeald a relevant message for the local physicians to keep in mind when managing patients with higher BMI during cardiac arrest.**

***Answers*** *We have added this information on page 10, lines 197-199.*
